# Developing countries and neglected diseases: challenges and perspectives

**DOI:** 10.1186/1475-9276-6-20

**Published:** 2007-11-26

**Authors:** Abdesslam Boutayeb

**Affiliations:** 1 UFR Modelling and Data Analysis, Faculty of Sciences-University Mohamed Ier, Oujda, Morocco

## Abstract

It is now commonly admitted that the so-called (most) neglected tropical diseases have been given little attention. According to World Health Organization, neglected diseases are hidden diseases as they affect almost exclusively extremely poor populations living in remote areas beyond the reach of health service. The European Parliament recognised that, to our shame, Neglected Diseases have not received the attention they deserve from EU actions. In the Millennium Development Goals they were given very little attention and mentioned just as other disease. Investing in drugs for these diseases is thought to be not marketable or profitable. However, despite their low mortality, neglected diseases are causing severe and permanent disabilities and deformities affecting approximately 1 billion people in the world, yielding more than 20 millions of Disability Adjusted Life Years (56.6 million according to Lancet's revised estimates) and important socio-economic losses. Urgent pragmatic and efficient measures are needed both at international and national levels.

## 1. Introduction

At the dawn of the third millennium, while human rights and health equity are on all international agendas, millions of forgotten people are suffering from a dozen of neglected diseases (NDs). According to The World Health Organization (WHO), NDs are hidden diseases as they affect almost exclusively extremely poor populations living in remote areas beyond the reach of health services [1]. The European Parliament recognised that "to our shame, Neglected Diseases have not received the attention they deserve from EU actions" [2]. Focusing on the "big killers" like HIV/AIDS, malaria and tuberculosis, the Millennium Development Goals (MDG) and other initiatives have generally given very little attention to the most neglected diseases, often mentioned just as "other disease" (Table 1)[3]. Criticizing the "inertia" and the delay taken in the response to the infectious diseases, the humanitarian organization Médecins sans Frontière (MSF) has been continuously attracting the international attention to stimulate more interest in the development and provision of treatments for the most neglected diseases [4]. Meanwhile, beyond mortality figures, NDs continue to cause severe and permanent disabilities and deformities affecting more than a billion people in the world and breeding millions of disability adjusted life years (DALYs) and important economic losses. Indeed, lymphatic filariasis(LF), leishmaniasis, schistosomiasis, Buruli ulcer, cholera, cysticercosis, dracunculiasis (guinea-worm disease), foodborne trematode infections, hydatidosis, soil-transmitted helminthiasis (ascariasis, trichuriasis, hookworm diseases), trachoma, trypanosomiasis (sleeping sickness), onchocerciasis, Chagas disease, dengue and others [Additional file 1] are responsible for impaired childhood growth, mental retardation, blindness, amputation and diverse disability conditions and hence they are impeding human development of many countries of Africa and Latin America (Tables 2 and 3)[1,5-8]. The situation being commonly admitted, it remains that urgent and efficient strategies are needed at local, national and international levels in order to reduce the growing burden of these diseases of the poor.

**Table 1:** The Millennium Project [4]

Millennium Development Goals	UN Millennium Project task forces
1. Reduce extreme poverty and hunger by half relative to 1990	1. Poverty and economic development
2. Achieve universal primary education	2. Hunger
3. Promote gender equality & empowerment of women	3. Education and gender equality
4. Reduce child mortality by two-third relative to 1990	4. Child and maternal health
5. Improve maternal health, including reducing maternal mortality by three-quarters relative to 1990	5. HIV/AIDS, malaria, tuberculosis, and access to essential medicines
6. Prevent spread of HIV/AIDS, malaria, and other diseases	6. Environmental sustainability
7. Ensure environment sustainability	7. Water and sanitation
8. Develop a global partnership for development	8. Improving the lives of slum dwellers 9. Trade 10. Science, technology, and innovation

**Table 2:** The burden of neglected diseases according to WHO Report 2002 [1]

**Disease**	**Deaths**	**Burden in DALYs**
Lymphatic filariasis	0	5 654 000
Soil-transmitted helminthiasis	12 000	4 706 000
Kala-azar		2 357 000
Trachoma	0	2 329 000
Leishmaniasis	51 000	2 400 000
Schistosomiasis	15 000	1 760 000
Sleeping sickness	48 000	1 600 000
Onchocerciasis	0	987 000
Dengue		700 000
Chagas disease	14 000	649 000
Leprosy	6 000	177 000
Buruli ulcer		100 000
Guina-worm		100 000

**Table 3:** The burden of neglected diseases revised estimates (The Lancet) [6]

**Disease**	**Deaths**	**Burden in DALYs (in million)**
Hookworm diseases		22.1
Ascariasis		10.5
Trichuriasis		6.4
Lymphatic filariasis	0	5.8
Trachoma	0	2.3
Leishmaniasis	100 000	2.1
Schistosomiasis	150 000 -200 000	4.5
Sleeping sickness	100 000	1.5
Onchocerciasis	0	0.5
Chagas disease	14 000	0.7
Leprosy	6 000	0.2
Buruli ulcer		NA

Total	500 000	56.6

## 2. Neglected diseases afflicting marginalised populations: Challenges and perspectives

Neglected Diseases are given low priority because they have low mortality, they occur almost exclusively in poor developing countries and essentially, because they offer negligible marketable and profitable issues. As stressed by the European Parliament Report in 2005, "No research is currently being carried out into the most neglected diseases which mainly affect developing countries...there is a chronic shortage of investment in research and development in poverty-related diseases and in the developing countries themselves to obtain medicines which meet the needs of those countries" [2].

For the pharmaceutical industry, which carries out the main research and development for new drugs, it is too costly and risky to invest in drugs for neglected diseases occurring essentially in low-income countries where public spending on drugs is less than US$6 (sub-Saharan Africa) compared to around US$ 240 spent in countries of the Organization for Economic Cooperation and Development (OECD) [9]. It is estimated that, less than 10% of the world's biomedical research funds are dedicated to problems dealing with 90% of the world's burden of disease and, of all drugs in development for all neglected diseases in 1999–2000, 18 R&D projects were clinical development, compared to 2100 compounds for all other diseases [1,2]. Between 1975 and 2004, among the 1556 new molecules of drugs marketed in the world, only 21 were intended for the neglected diseases (8 for malaria, 3 for tuberculosis and only 10 for the whole set of most neglected diseases)[4]. Another study found that, of the 1393 new chemical entities marketed between 1975 and 1999, only 16 were for tropical diseases and tuberculosis, yielding a 13-fold greater chance for a drug to be marketed for central-nervous-system disorders or cancer than for a neglected disease (Table 4) [9].

**Table 4:** New chemical entities (NCEs) approved between 1975 and 1999 by drug class and relative to disease burden and drug sales [9]

**Therapeutic areas**	**Approved NCEs 1975–1999**	**Proportion of worldwide sales Year 1999**	**NCEs by DALY**	**Drug sales (millions of US$) by DALY**
Central nervous system	211 (15·1%)	15·1%	1·32	193
Cardiovascular	179 (12·8%)	19·8%	1·25	283
Cytostatics (neoplasms)	111 (8·0%)	3·7%	1·31	90
Respiratory (non-infectious)	89 (6·4%)	9·3%	1·44	307
Anti-infectives and antiparasitics	224 (16·1%)	10·3%	0·55	52
HIV/AIDS	26 (1·9%)	1·5%	0·37	44
Tuberculosis	3 (0·2%)	0·2%	0·11	11
Tropical diseases (Total)	13 (0·9%)	0·2%	0·10	3
Malaria	4 (0·3%)	0·1%	0·10	5
Other therapeutic categories	579 (41·6%)	41·9%	1·10	163

Total	1393 (100%)	100%	1·01	148

Despite the dilemma created by the pharmaceutical industry (treatment-profit), the responsibility is shared by other decision makers. At the global level, international solidarity and public-private partnerships are needed to tackle the problems of shortage and lack of treatments, resistance and the need for new drugs and vaccines. More initiatives are needed to support the projects already launched such as Global Alliance for Vaccines and Immunization (GAVI), The Human Hookworm Vaccine initiative (HHVI), the Foundation for Innovative New Diagnostics (FIND), the Drug for Neglected Diseases Initiative (DNDi), The USAID funded program on integrated control of seven of the most prevalent neglected tropical diseases (trachoma, hookworm, ascariasis, trichuriasis, onchocerciasis, schistosomiasis and lymphatic filariasis) and others [1,2,10-12].

However, this international strategy is insufficient without the national and local implication. National health decision makers, non governmental organizations (NGOs), research institutions, community groups and individuals must adhere to these global initiatives. Many countries, being heavily indebted, affect less than 1% of the national global budget to health, and few governments are putting science, technology, and innovation at the centre of their strategies and, in the meantime, war and conflicts are financed at the expense of health services. For instance, in 1999, the governments of sub-Saharan Africa dedicated US$7 billion to military spending, whereas, diverting just 15% of this would have raised more than one US$ billion, enough to treat millions of patients affected by neglected diseases[13,14]. It is also worth stressing that, in the absence of reporting and surveillance, the available statistics on the burden of NDs are sometimes very different as indicated in Tables 2 and 3.

To overcome this odd situation and in order to reduce the burden of neglected diseases afflicting mainly poor populations of developing countries, pragmatic and efficient strategies are urgently needed. Beside large campaigns for education and sensitisation, measures may include advance purchase commitments, tax credits, fee waivers, partial transfer of patent rights, innovation prizes, technology transfer, health innovation and various incentives for investment. These would promote development of drugs and vaccines for neglected diseases by enhancing collaboration with the pharmaceutical industry of developed countries and encouraging research and development for drugs in developing countries [1-5,10-15].

## 3. Conclusion

In many developing countries, millions of people live with less than one dollar a day and on fragile and often remote rural ecosystems, most of them lack access to basic health services and safe drinking water and sanitation (vectors that transmit NDs thrive on these ideal conditions). Trapped in the vicious circle of underdevelopment-poverty-health inequity, these populations constitute exhausted "preys" for "predators" such as HIV/AIDS, malaria, tuberculosis and a multitude of the so-called neglected diseases. The growing attention given to the "Big Three Killers" should not shadow the suffering that neglected diseases are causing to millions of people who can afford, at best, archaic drugs, some of which are toxic, ineffective or difficult to administer.

In the era of science and high technology, while regular meetings are held worldwide to discuss human rights and various forms, causes and consequences of health inequities, it is a shame that poor populations living in developing countries are denied access to adequate and affordable treatment against NDs. Urgent actions are needed to develop new drugs and vaccines that are efficient and accessible. A big challenge is addressed to national and international decision makers but it is worth trying.

## Competing interests

The author(s) declare that they have no competing interests.

## Supplementary Material

Additional file 1 An Overview of Neglected Diseases Impact. Neglected diseases such as lymphatic filariasis, leishmaniasis, schistosomiasis, sleeping sickness, Chagas disease, Buruli ulcer, dengue and others are responsible for impaired childhood growth, mental retardation, blindness, amputation and diverse disability conditions. A brief overview of these diseases and their impact is given in this file. Click here for file
