# Supplementary material for: Developing countries and neglected diseases: challenges and perspectives
Source: Int J Equity Health. 2007 Nov 26;6:20. doi: 10.1186/1475-9276-6-20 (PMC2206025; doi:10.1186/1475-9276-6-20)
Supplement: Additional file 1 — An Overview of Neglected Diseases Impact. Neglected diseases such as lymphatic filariasis, leishmaniasis, schistosomiasis, sleeping sickness, Chagas disease, Buruli ulcer, dengue and others are responsible for impaired childhood growth, mental retardation, blindness, amputation and diverse disability conditions. A brief overview of these diseases and their impact is given in this file. [file 1475-9276-6-20-S1.doc]

An Overview of Neglected Diseases Impact

Abdesslam Boutayeb

Beyond mortality figures, the health impact of neglected diseases (NDs) can be measured by severe and permanent disabilities and deformities affecting approximately 1 billion people in the world and causing millions of Disability Adjusted Life Years (DALYs), mainly in Africa and Latin America. Indeed, lymphatic filariasis (LF), leishmaniasis, schistosomiasis, sleeping sickness, Chaga disease, Buruli ulcer, dengue and others are responsible for impaired childhood growth, mental retardation, blindness, amputation and diverse disability conditions. A brief overview, essentially based on the estimates of the World Health Organization (WHO), is given below.

**1. Lymphatic Filariasis (LF)**

During centuries, this disease, also known as Elephantiasis because it results in the grossly enlarged limbs, has been causing severe disability and immense suffering. Endemic in over 80 countries, where 120 million people are infected and more than a billion people are at risk, LF is currently ranking as the second leading cause of disability, seriously incapacitating at least 40 million people worldwide. African and Asian populations living in Least-developed, low-income and lower-middle-income countries represent nearly 100% of people at risk for LF. Gains following LF elimination are expected to approach US$4 billion per year.

Treatment is by a once-yearly administration of single doses of albendazole plus either dietthylcarbamazine or ivermectin carried out for 4-6 years.

### 2. Leishmaniasis

According to WHO estimates, this disease is endemic in 88 countries, where 12 million people are currently affected and 350 million people are at risk. However, as a disease of poverty that causes high morbidity but low mortality, its burden remains difficult to evaluate. The disease thrives in impoverished urban areas and refugee camps, and malnutrition is a well-known risk factor for visceral leishmaniasis in particular. Sudan is the African country which has the most suffered from this disease.

About 1.5 to 2 million new cases occur annually with a rising trend.

The most life-threatening form is Viscereal Leishmaniasis (VL) (kala-azar) which attacks the immune system from which 57 000 deaths were reported in 1999.

The most widely prescribed drug to treat this disease is pentavalent antimony, discovered a century ago. The drug has serious side effects, needs prolonged treatment and is loosing efficacy in some regions due to parasite resistance. Newer treatment have problems of toxicity, high price, and/or difficulty of administration.

It should be stressed that AIDS and VL reinforce one other and co-infection poses additional problems. AIDS increases the risk of VL by 100-1000 times in endemic areas.

**3. Schistosomiasis and soil-transmitted hemlinthiasis (hookworm, ascariasis, trichuriasis)**

Poor populations are suffering from these ancient diseases that cause misery and disability to millions of people. It is thought that 2 billion people are affected worldwide, of whom 300 million suffer severe morbidity. In 1999, WHO estimated that these two diseases represented more than 40% of the disease burden due to all tropical diseases, excluding malaria. Schistosomiasis is known to cause not only forms of morbidity but also irreversible damage to the liver, kidney and urinary tract. Despite the success of ongoing control programmes in certain Asian and North African countries, where mortality, morbidity and transmission have been reduced, the global number of infected people has not changed for 50 years, mainly due to dramatic increases in sub-Saharan Africa. Indeed, in 2000, 85% of all people affected by this disease were living on the African continent. Schistosomiasis (bilharzia) alone infects 200 million people, causing suffering with severe consequences, causing about 20 000 deaths annually.

Treatment with praziquantel exists but the emergence of resistant parasites may complicate the task. There is a need for alternative therapies.

4. African trypanosomiasis (sleeping sickness)

This old parasitic disease is now endemic, with a growing prevalence, in 36 sub-Saharan African countries where 60 million people are at risk. An estimated 300 000 to 500 000 people are thought to be infected. Most of them are living in remote rural areas on less than US$150 per year. The disease is having devastating consequences on the development in Africa. It causes over 3 million livestock deaths each year and an annual loss of US$ 4.5 billion in agriculture.

The urgent need for developing new treatment is justified by the limited choice of available drugs, their toxicity (causing mortality in 5% of patients) and resistance.

### 5. American trypanosomiasis (Chaga's disease)

Chaga's disease is prevalent in Central and South America where 25% of the population is at risk. An estimated 18 million people are infected by the parasite and 50 000 deaths occur annually. Only two drugs (nifurtimox and benznidazole) exist but there is no treatment for chronic Chaga's disease.

**6. Dengue**

A re-emergent disease, endemic in more than 100 countries. Its incidence has increased fourfold since 1970 and nearly half the world’s population (2.5-3 billion) is at risk. It is estimated that more than 50 million people are infected annually of which a million are affected by the more severe form Dengue Haemorrhagic Fever. WHO estimates that dengue causes a burden of 0.7 million DALYs but according to other sources**,** the burden is much higher. No treatment or vaccine is available at the moment.

### 7. Buruli ulcer

Buruli ulcer is a severely and disabling bacterial disease reported from 30 countries throughout the tropical world. According to WHO, West Africa is by far the most affected region. In some communities in Ghana and Côte d’Ivoire, with a prevalence reaching 20%, the disease is now the second most prevalent myocobacterial disease after tuberculosis. The disease can be cured if detected early. However, in Africa, less than 10% of cases are seen at this stage. Consequently, most infected people will end up with irreversible deformity. The disease is most common in children under the age of 15, who account for 70% of all cases. With an average cost of treatment estimated at US$780 per patient, this disease exacerbates health problems in many African countries.

At present, the only treatment available is surgery to remove the lesion and a skin graft if necessary. This is both costly and dangerous, with loss of tissues or permanent disability.

8. Trachoma

Worldwide, nearly 10% of the population is at risk of blindness from trachoma, but its impact goes beyond vision, it affects seriously productivity and development in general.

9. Leprosy

According to WHO, 14.5 million patients have been cured from leprosy since 1985, through multidrug therapy. Today, less than a million people are affected by the disease.

10. Guinea-worm disease (dracunculiasis)

Before eradication programme started in early 1980s, about 3.5 million people in 20 endemic countries were infected. In 2005, only about 10 000 cases were reported in 9 endemic countries

11. Onchocerciasis

More than 25 million hectares of land available for settlement and agricultural cultivation which are now disease-free, improving thereby the development in Africa and Latin America, were previously infected.

### 12. Neuropsychatric disorders

Mental illnesses are affecting all countries with an increasing trend. Epilepsy affects 50 million people of which 80% in developing countries. It is thought that at least 70% of people affected can be seizure-free treated with antiepileptic drugs. However, the vast majority remain untreated (in Africa, 80% receive no treatment).

**13. Other diseases:**

Although with less impact, other NDs are affecting poor populations in developing countries. These include cholera, cysticercosis, foodborne trematode infections and hydatidosis.
